# Concordance between Different Criteria for Metabolic Syndrome in Peruvian Adults Undergoing Bariatric Surgery

**DOI:** 10.3390/jcm11164692

**Published:** 2022-08-11

**Authors:** Nataly Echevarria-Castro, Kevin Silva-Parra, Marcos Polar-Trinidad, Juan C. Sánchez-Vicente, Gustavo Salinas-Sedo, Carlos J. Toro-Huamanchumo

**Affiliations:** 1 Facultad de Ciencias de la Salud, Universidad Peruana de Ciencias Aplicadas, Lima 15067, Peru; 2 Facultad de Medicina Humana, Universidad Nacional San Luis Gonzaga, Ica 11004, Peru; 3 Unidad de Investigación Multidisciplinaria, Clínica Avendaño, Lima 15074, Peru; 4 Unidad de Investigación para la Generación y Síntesis de Evidencias en Salud, Universidad San Ignacio de Loyola, Lima 15024, Peru

**Keywords:** metabolic syndrome, obesity, bariatric surgery, latinos, adults

## Abstract

Background: Metabolic Syndrome (MetS) is a clinical entity that has been linked to several non-communicable diseases. There are various consensuses to determine its presence, such as the IDF, ALAD, Harmonized, AHA/NHLBI, NCEP-ATP III or AACE criteria. However, there is currently no standardization to properly identify it. Objective: To assess the diagnostic concordance between different criteria for MetS in Peruvian adults undergoing bariatric surgery. Methods: We conducted a secondary analysis of the institutional database of a bariatric clinic located in Lima, Peru. We obtained data from adults between 18–59 years who underwent bariatric surgery (Roux-en-Y Gastric Bypass or Sleeve Gastrectomy). According to the Kappa coefficient, a heatplot was designed to analyze the concordance of the criteria. Results: An almost perfect concordance was found between all criteria except AACE. The highest kappa coefficient (κ = 0.980) was recorded between the IDF and ALAD criteria using all the sample. Similar results were obtained when we stratified by sex. Conclusions: This study shows that, excluding the AACE, different criteria for metabolic syndrome could be used in Latino adults undergoing bariatric surgery with similar results. Given the postoperative implications, we believe that IDF and ALAD would be the best options in our population.

## 1. Introduction

Metabolic Syndrome (MetS) is an entity that comprises multiple abnormal metabolic conditions [1] linked with increased cardiovascular risk [2]. Some related diseases include coronary heart disease, type 2 diabetes mellitus (T2DM), polycystic ovary syndrome, non-alcoholic fatty liver (NAFLD), asthma, sleep disorders and some types of cancer [3,4]. Approximately a quarter of world′s population has MetS [5].

It is essential to properly identify patients with MetS in order to provide early treatment and prevent unwanted clinical outcomes [6]. The treatment of MetS includes lifestyle changes, pharmacological management and surgical therapies such as bariatric surgery (BS) [1]. BS causes weight loss by different procedures that include gastric volume restriction and malabsorption [7]. Hence, as one of the objectives of BS is the resolution of MetS, it is essential to reach a standardized definition in this population [8].

Multiple studies have evaluated MetS in people who have undergone BS, each with different diagnostic criteria [9,10,11]. In recent years, there has been increased use of the International Diabetes Federation (IDF) definition for MetS, especially in the European adult population [8,12]. Other studies in Hispanic adults have used the definition provided by the Harmonized criteria, in which the elevated waist circumference (WC) is replaced by a body mass index (BMI) ≥ 30 kg/m^2^ [13]. Finally, few studies use the definition provided by the National Cholesterol Education Program (NCEP) Adult Treatment Panel III (ATP-III), research conducted in the United States population, which uses a highest cut-off value for WC as a necessary factor [14,15].

The Latino population has epidemiological and genetic factors linked to elevated MetS incidence. These include ethnicity, malnutrition during childhood, maternal malnutrition, high carbohydrate diet, aging population and a high prevalence of insulin resistance [16,17]. Latin America reports a prevalence of MetS of 25.3% for women and 23.2% for men, higher than other regions [17,18,19]. In Peru, cross-sectional studies using International Diabetes Federation (IDF) criteria report a prevalence of approximately 30% [20,21]. Due to the lack of epidemiological data and differences in criteria used, the consensus of the Latin American Diabetes Association (ALAD, by its acronym in Spanish) was carried out in 2010 and proposed five clinical and laboratory values for the diagnosis of MetS [16], based on diverse epidemiologic and clinical factors recommended by reviewing published literature from Latin American countries [16]. However, to date, there are no studies evaluating concordance between ALAD and other criteria for MetS [22,23].

This study aimed to evaluate the diagnostic concordance between six different criteria for MetS in Peruvian adults undergoing bariatric surgery.

## 2. Materials and Methods

### 2.1. Study Design and Population

This was a secondary analysis that used the institutional database from a bariatric clinic located in Lima, Peru. We included data from 18–59 years old adults who underwent bariatric surgery (either Roux-en-Y Gastric Bypass (RYGB) or Sleeve Gastrectomy (SG)). Hence, we included: (a) patients with BMI ≥ 30 kg/m^2^ who had unsuccessfully tried to lose weight with lifestyles or pharmacological methods and T2DM and/or poorly controlled arterial hypertension in spite of optimal medical therapy; (b) patients with BMI ≥ 35 kg/m^2^ who had unsuccessfully tried to lose weight with lifestyles or pharmacological methods and T2DM, hypertension, sleep apnea NAFLD and/or other poorly controlled comorbidities secondary to obesity in spite of optimal medical therapy; (c) patients with BMI ≥ 40 kg/m^2^, and; (d) patients (selected on a case-by-case basis) with NAFLD and/or insulin resistance with an inability to achieve a healthy weight loss sustained for a period of time with prior non-surgical weight loss methods.

### 2.2. Criteria for Metabolic Syndrome 

We considered six different criteria for MetS that were the most commonly used in the current literature (Table 1):

Latin American Consensus of the Latin American Diabetes Association (ALAD): MetS was defined by the presence of abdominal obesity: WC ≥ 94 cm (men) and ≥88 cm (women), plus two of the following four conditions: Triglycerides (TG) > 150 mg/dL or on hypertriglyceridemia treatment, High-density lipoprotein cholesterol (HDL-C) < 40 mg/dL (men) and < 50 mg/dL (women) or on treatment for low HDL-C, high blood pressure (BP) as systolic blood pressure (SBP) ≥ 130 mmHg and/or diastolic blood pressure (DBP) ≥ 85 mmHg or on antihypertensive drugs, impaired fasting glucose (IFG)/impaired glucose tolerance (IGT) or diabetes [16].

Harmonized criteria: MetS was defined by the presence of three of these five conditions: increased WC depending on the population/country, TG ≥ 150 mg/dL or on hypertriglyceridemia treatment, HDL-C < 40 mg/dL (men) and <50 mg/dL (women) or on treatment for low HDL-C, SBP ≥ 130 mmHg and/or DBP ≥ 85 mmHg or on antihypertensive treatment, fasting plasmatic glucose (FGP) ≥ 100 mg/dL or on hyperglycemia treatment [24].

International Diabetes Federation (IDF): MetS was defined by a WC ≥ 90 cm for males and WC ≥ 80 for females plus at least two of the following four conditions: TG > 150 mg/dL or on treatment for this lipid abnormality, HDL-C < 40 mg/dL (men) and <50 mg/dL (women) or on treatment for reduced HDL-C, SBP ≥ 130 and/or DBP ≥ 85 mmHg or on treatment for hypertension, raised FPG ≥ 100 mg/dL or previously diagnosed T2DM [25].

National Cholesterol Education Program-Adult Treatment Panel III (NCEP-ATPIII): MetS was defined by the presence of three or more of the following five conditions: WC > 102 cm (men) and > 88 cm (women), TG ≥ 150 mg/dL, HDL-C < 40 mg/dL (men) and <50 mg/dL (women); SBP ≥ 130 and DBP ≥ 85 mmHg or on antihypertensive treatment and IFG ≥ 110 mg/dL [2].

American Heart Association/National Heart, Lung, and Blood Institute Scientific Statement (AHA/NHLB): MetS was defined by the presence of three of these five conditions: WC ≥ 102 cm (men) and ≥ 88 cm (women), TG ≥ 150 mg/dL or on hypertriglyceridemia treatment, HDL-C < 40 mg/dL (men) and < 50 mg/dL (women) or on treatment for reduced HDL-C, SBP ≥ 130 mm Hg and/or DBP ≥ 85 mm Hg or on antihypertensive treatment, fasting glucose ≥ 100 mg/dL or on hyperglycemia treatment [4].

American Association of Clinical Endocrinologists (AACE): Clinical definition of metabolic syndrome depends on clinical judgment according to the presence of BMI ≥ 25 kg/m^2^, TG ≥ 150 mg/dL and HDL-C < 40 mg/dL (men) and < 50 mg/dL (women), BP ≥ 130/85 mmHg or antihypertensive treatment, 2-h post glucose challenge > 140 mg/dL, Fasting glucose 110–126 mg/dL. The AACE criteria does not considerate treatment for specific diseases except for hypertension. Additional risk factors include a family history of T2DM, hypertension, coronary heart disease (CHD), polycystic ovary syndrome (PCOS), sedentary lifestyle, advanced age, ethnic groups at high risk for T2DM or for CHD [26].

### 2.3. Other Variables 

We considered other variables such as sex (male or female), age (in years), weight (in kilograms), height (in meters), BMI (in kg/m^2^), SBP and DBP (both in mmHg), fasting glucose (in mg/dL), HDL-C (in mg/dL), TG (in mg/dL), insulin (in uU/dL), HOMA-IR and WC (in cm).

### 2.4. Statistical Analysis

We presented categorical data as frequencies and percentages (%) and numerical data with mean and standard deviation (SD) or median and interquartile range (IQR), according to the distribution. Student′s *t*-test or Mann–Whitney U test was used to assess significant differences between baseline characteristics according to sex.

Kappa (k) statistic was used for assessing the agreement between the different criteria of MetS and a heatplot was designed. *p*-values < 0.05 were considered significant, and all the analyses were performed using the statistical package Stata v15.1 (StataCorp, College Station, TX, USA).

### 2.5. Ethics

The present study was approved by the Institutional Review Board of the Clínica Avendaño. Participant consent was not required, and the study database was de-identified.

## 3. Results

### 3.1. Characteristics of Participants

In total, 205 participants met the inclusion criteria and were enrolled in this study. The 62.9% (n = 127) were women and the median BMI was 37.44 kg/m^2^ (140 and 65 patients had a BMI < 40 kg/m^2^ and ≥40 kg/m^2^, respectively). In addition, 15 (7.3%), 37 (18.1%) and 10 (4.9%) reported a history of T2DM, hypertension, and obstructive sleep apnea that had not responded effectively to medical therapy. NAFLD and insulin resistance were present in almost all (>90%) of the participants.

A higher value was found for weight (*p* < 0.001), height (*p* < 0.001), BMI (*p* < 0.001), systolic blood pressure (*p* < 0.001), diastolic blood pressure (*p* < 0.001), triglycerides (*p* = 0.002), insulin (*p* < 0.001), HOMA-IR (*p* < 0.001), waist circumference (*p* < 0.001) in men and a higher HDL cholesterol value (*p* < 0.001) in women. Sociodemographic characteristics and components of Mets are detailed in Table 2.

### 3.2. Metabolic Syndrome Diagnosis Criteria

Table 3 shows that more than 50% of the participants had MetS according to all the metabolic syndrome criteria, except for the AACE criteria. MetS was present in 59.5% (IDF), 58.5% (ALAD), 55.6% (Harmonized), 54.2% (AHA/NHLBI) and 50.7% (NCEP-ATP III) of the population. AACE was the only criteria with a frequency lower than 25% of the studied population (22.9%).

### 3.3. Concordance of Metabolic Syndrome Definitions

**Figure 1:**
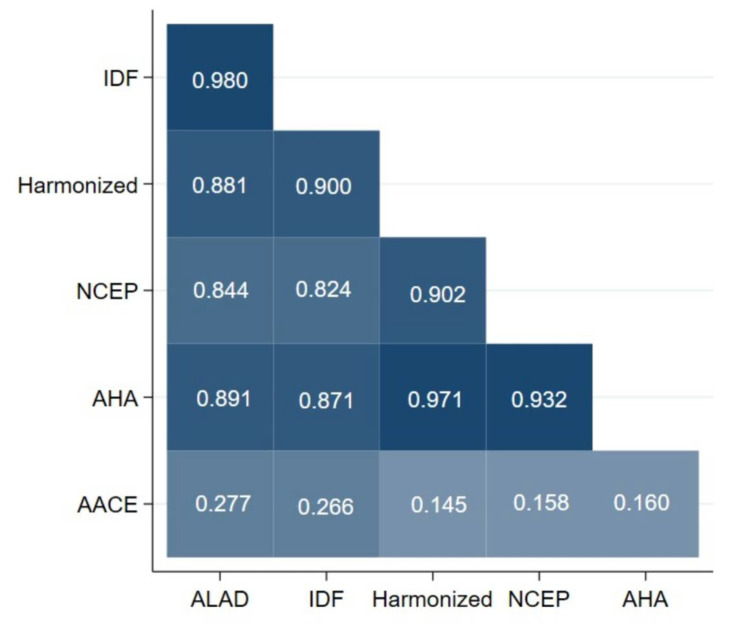
Heatplot of metabolic syndrome diagnostic criteria in the study population. ALAD: Latin American Consensus of the Latin American Diabetes Association; IDF: International Diabetes Federation; NCEP-ATP III: National Cholesterol Education Program-Adult Treatment Panel III; AHA/NHLB: American Heart Association/National Heart, Lung, and Blood Institute Scientific Statement; AACE: American Association of Clinical Endocrinologists.

In Figure 1, we presented a heatplot designed to assess the concordance between the different MetS criteria. An almost perfect concordance was found between all criteria except the AACE criteria. The highest kappa coefficients were recorded between the IDF and ALAD (κ = 0.980), AHA/NHLBI and Harmonized criteria (κ = 0.971), and AHA/NHLBI and NCEP-ATP III (κ = 0.932). The criteria that showed the least concordance with the others was AACE.

**Figure 2:**
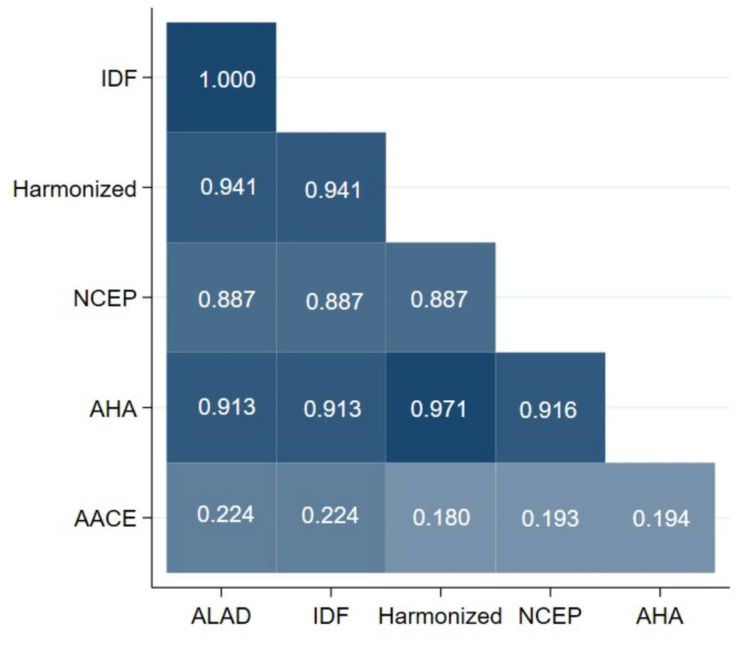
Heatplot of metabolic syndrome diagnostic criteria in men. ALAD: Latin American Consensus of the Latin American Diabetes Association; IDF: International Diabetes Federation; NCEP-ATP III: National Cholesterol Education Program-Adult Treatment Panel III; AHA/NHLB: American Heart Association/National Heart, Lung, and Blood Institute Scientific Statement; AACE: American Association of Clinical Endocrinologists.

In Figure 2, we present a heatplot of MetS diagnostic criteria in men. A perfect concordance was found between IDF and ALAD (κ = 1). An almost perfect concordance was found between AHA and Harmonized (κ = 0.971); AHA and NCEP-ATP III (κ = 0.916), and; Harmonized with ALAD and IDF (κ = 0.941). Similar to Figure 1, AACE criteria showed the weakest concordance with other criteria.

**Figure 3:**
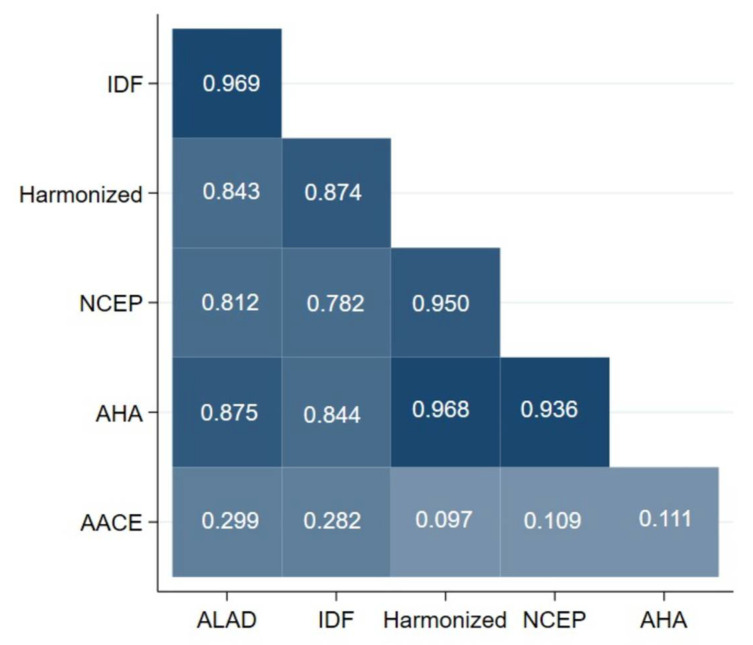
Heatplot of metabolic syndrome diagnostic criteria in women. ALAD: Latin American Consensus of the Latin American Diabetes Association; IDF: International Diabetes Federation; NCEP-ATP III: National Cholesterol Education Program-Adult Treatment Panel III; AHA/NHLB: American Heart Association/National Heart, Lung, and Blood Institute Scientific Statement; AACE: American Association of Clinical Endocrinologists.

In Figure 3, we show heatplot of MetS diagnostic criteria in women. The most important results were the concordance between IDF and ALAD (κ = 0.969), NCEP-ATP III and Harmonized (κ = 0.950), AHA and Harmonized (κ = 0.968) and AHA with NCEP-ATP III (κ = 0.936). Similar to Figure 2 and Figure 3, AACE criteria showed the weakest concordance with other criteria.

## 4. Discussion

### 4.1. Main Findings

This study was conducted in adults undergoing bariatric surgery to assess the concordance between the criteria for metabolic syndrome. We found an almost perfect concordance between IDF/ALAD/Harmonized/AHA/NHLBI/NCEP-ATP III criteria, regardless of the sex of the study subject. The highest concordance in the study was found between ALAD and IDF.

### 4.2. Comparison with Other Studies

The lowest prevalence of metabolic syndrome was found with the AACE criteria (22.9%) and the highest prevalence was found with the IDF criteria (59.5%). In Peru, a previous study among adults with overweight and obesity reported the prevalence of MetS using different criteria. The lowest prevalence of MetS in this study was found with the WHO criteria (42%) and the highest prevalence was found with the Szabo criteria (74.3%). Comparing their results with ours, according to the criteria, we found the following prevalence of MetS: IDF (58.6% vs. 59.5%), AHA / NHLBI (52.9 % vs. 54.1%) and NCP-ATP (56% vs. 50.7%) [23]. Another study conducted by Vasquez MA et al. (2016) in Ecuador, found that the concordance between IDF and ALAD criteria was almost perfect, as in our study [27].

The most frequent criteria used in epidemiological and clinical studies in Peru are IDF, ALAD and NCP-ATP III [20,21,28]. In our study, we found an almost perfect concordance between these criteria. A similar concordance was founded in a previous study conducted in Peruvian population, although they did not consider the ALAD criteria [23]. 

### 4.3. Results Interpretation

Current research confirmed the concordance between ALAD and IDF criteria, possibly due to the indispensable use of the waist circumference as criterion, unlike the other criteria. Likewise, the cut-off points of the waist circumference are relatively similar, mainly in men [16,25]. In our study, AACE was the one with the weakest level of concordance with the others, possibly due to the fact that IFG or IGT are indispensable or that AACE does not considered actual treatment for lipid abnormalities or hyperglycemia [26]. Our database had fasting glucose values; however, we do not have values from the oral glucose-tolerance test; so we cannot detect if the patient has IGT. Therefore, it could not be correctly assessed whether patients meet the AACE criteria.

### 4.4. Relevance in Public Health and Clinical Practice

Metabolic syndrome is characterized by a progressive deterioration of quality of life and a strong association with multiple diseases such as T2DM, hypertension, dyslipidemia and obesity [29]. Moreover, less labor productivity and absenteeism associated with this condition contribute to a detriment of the family and personal economy [29]. The prevalence of MetS is increasing worldwide, especially in recent years [30]. In Latin America, the prevalence of MetS varies by city, the lowest prevalence is reported in Quito (13.7%) and the highest in Mexico City (27.0%) [31]. We did not find national representative data from Peru.

As previously mentioned, there is high concordance between the evaluated criteria, with the exception of the ACEE criteria. Thus, in the adult population undergoing bariatric surgery and, by extrapolation, in the population of adults with obesity/overweight, could make sense to replace one criteria of metabolic syndrome with another in the case of not being able to use one of them. This recommendation is mainly between the IDF and ALAD, AHA and Harmonized, and AHA and NCEP-ATP III criteria. Specifically, since adults with MetS have an increased risk of morbidity and mortality after bariatric surgery [14], sufficiently sensitive criteria should be used for their accurate diagnosis. In this sense, ALAD and IDF could be the best choices for Latin American adults undergoing bariatric procedures.

### 4.5. Limitations

The study had some limitations. First, the study cannot be extrapolated to adolescents or older adults since only participants between 18–59 years of age who underwent bariatric surgery were considered; however, we consider that our results can be extrapolated to the population of adults with overweight/obesity who are candidates for bariatric surgery. Second, there is a possibility that some variables were mismeasured. However, the personnel that filled the database received intensive training on the correct filling of the base. We also conducted a rigorous assessment of the data quality, which consisted of identifying missing and implausible data, in addition to independent double coding and cross-checking of the databases, in order to reduce the possibility of information bias. Third, the absence of variables such as oral glucose tolerance, family history of DM2 and polycystic ovary syndrome might have limited the assessment of the AACE criteria.

## 5. Conclusions

This study shows that excluding the AACE, different criteria for metabolic syndrome could be used in Latino adults undergoing bariatric surgery with similar results. Given the postoperative implications, we believe that IDF and ALAD would be the best options for our population, although further studies might be necessary to extrapolate our results to similar populations from other Latin American countries. Similarly, future cohort design studies should compare the concordance of these criteria in the middle- and long-term follow-up (i.e., assessing the resolution of MetS after bariatric surgery).

## Figures and Tables

**Table 1:** Definitions of metabolic syndrome.

Measure	Metabolic Syndrome Criteria					
	ALAD	Harmonized	IDF	NCEP-ATP III	AHA/NHLBI	AACE
Diagnosis criteria	Abdominal obesity plus 2 of this 4	Any 3 of 5	Increased WC plus any 2 of this 4	Any 3 of this 5	any 3 of 5	IGT or IFG plus any of the following based on clinical judgment
Obesity	WC ≥ 94 cm (men) or ≥ 88 cm (women)	WC depends on the population/ country	WC with ethnicity specificity values	WC > 40 inches (men) and >35 inches (women)	WC > 40 inches (men) and >35 inches (women)	BMI ≥ 25 kg/m^2^
Dyslipidemia	TG > 150 mg/dL or Tx	TG ≥ 150 mg/dL or Tx	TG ≥ 150 mg/dL or Tx	TG ≥ 150 mg/dL or Tx	TG ≥ 150 mg/dL or T	TG ≥ 150 mg/dL and HDL-C < 40 mg/dL (men) and <50 mg/dL (women)
Dyslipidemia (second, separated criteria)	HDL-C < 40 mg/dL (men), <50 mg/dL (women) or Tx	HDL-C < 40 mg/dL (men) and <50 mg/dL (women) or Tx	HDL-C < 40 mg/dL (men) and <50 mg/dL (women) or Tx	HDL-C < 40 mg/dL (men) and <50 mg/dL (women) or Tx	HDL-C < 40 mg/dL (men) and <50 mg/dL (women) or Tx	
Blood pressure	SBP ≥ 130, DBP ≥ 85 mmHg, or Tx	SBP ≥ 130, DBP ≥ 85 mmHg, or Tx	SBP ≥ 130, DBP ≥ 85 mmHg, or Tx	SBP ≥ 130, DBP ≥ 85 mmHg, or Tx	SBP ≥ 130, DBP ≥ 85 mmHg, or Tx	SBP ≥ 130, DBP ≥ 85 mmHg or Tx
Glucose	IFG, IGT, or diabetes	Fasting glucose ≥ 100 mg/dL, or Tx	FPG ≥ 100 mg/dL or previously diagnosed T2DM	FPG ≥ 100 mg/dL or Tx	FPG ≥ 100 mg/dL or Tx	IGT or IFG (but not diabetes)
Other	-	-	-	-	-	other features of insulin resistance *

* Includes family history of type 2 diabetes mellitus, polycystic ovary syndrome, sedentary lifestyle, advancing age and ethnic groups susceptible to type 2 diabetes mellitus. WC: waist circumference. IFG: impaired fasting glucose. IGT: impaired glucose tolerance. TG: triglycerides. SBP: systolic blood pressure. DBP: diastolic blood pressure. BMI: body mass index. HDL-C: high-density lipoprotein cholesterol. FPG: fasting plasmatic glucose. Tx: current treatment for the specific component of MetS. ALAD: Latin American Consensus of the Latin American Diabetes Association. Harmonized: Harmonized criteria. IDF: International Diabetes Federation. NCEP-ATP III: National Cholesterol Education Program-Adult Treatment Panel III. AHA/NHLBI: American Heart Association/National Heart, Lung, and Blood Institute Scientific Statement. AACE: American Association of Clinical Endocrinologists.

**Table 2:** Sociodemographic data and components of metabolic syndrome.

Variable	Total (n = 205)	Male (n = 78)	Female (n = 127)	*p*
Age (years) *	36.7 ± 10.0	36.8 ± 9.9	36.7 ± 10.2	0.927 **
**Weight (kg) ^†^**	100.2 (89.8–115.6)	116.5 (104.0–131.3)	93 (85.4–101.7)	<0.001 ^††^
**Height (m) ^†^**	1.63 (1.58–1.72)	1.7 (1.7–1.8)	1.6 (1.5–1.6)	<0.001 ^††^
**BMI (kg/m^2^) ^†^**	37.44 (33.93–40.77)	39.4 (35.9–42.6)	36.3 (33.3–39.5)	<0.001 ^††^
**SBP (mmHg) ^†^**	120 (110.0–130.0)	124.5 (120.0–130.0)	117.0 (110.0–125.0)	<0.001 ^††^
**DBP (mmHg) ^†^**	80.0 (70–85)	80.0 (75.0–86.0)	75.0 (70.0–80.0)	<0.001 ^††^
**Glucose (mg/dL) ^†^**	92.0 (86.0–98.0)	93.5 (86.0–101.0)	92.0 (86.0–97.0)	0.254 ^††^
**HDL-C (mg/dL) ^†^**	41.0 (36.0–49.0)	37.5 (33.0–45.0)	44.0 (37.0–52.0)	<0.001 ^††^
**Triglycerides (mg/dL) ^†^**	154.0 (117.0–214.0)	174.0 (127.0–235.0)	143.0 (112.0–191.0)	0.002 ^††^
**Insulin (uU/mL) ^†^**	23.2 (15.9–29.6)	26.3 (19.4–37.9)	20.5 (15.0–27.3)	<0.001 ^††^
**HOMA-IR ^†^**	5.3 (3.6–7.3)	6.2 (4.5–9.1)	4.8 (3.2–6.6)	<0.001 ^††^
**WC (cm) ^†^**	113.0 (104.0–124.0)	123.0 (114.0–134.0)	106.0 (100.0–117.0)	<0.001 ^††^

* Mean ± Standard deviation; ^†^ Median (25th and 75th percentile); ** Student′s *t*-test. ^††^ Mann–Whitney U test. BMI: body mass index. SBP: systolic blood pressure. DBP: diastolic blood pressure. HDL-C: high-density lipoprotein cholesterol. HOMA-IR: Homeostatic Model Assessment for Insulin Resistance. WC: waist circumference.

**Table 3:** Frequency of metabolic syndrome based on different diagnostic criteria.

MetS Criteria	AACE n (%)		AHA/NHLBI n (%)		NCEP-ATP III n (%)		Harmonized n (%)		IDF n (%)		ALAD n (%)	
	Yes	No	Yes	No	Yes	No	Yes	No	Yes	No	Yes	No
Total	47 (22.9)	158 (77)	111 (54.1)	94 (45.9)	104 (50.7)	101 (49.3)	114 (55.6)	91 (44.4)	122 (59.5)	83 (40.5)	120 (58.5)	85 (41.5)
Male	22 (28.2)	56 (71.8)	52 (66.7)	26 (33.3)	49 (62.8)	29 (37.2)	53 (68)	25 (32.1)	53 (68)	25 (32.1)	53 (68)	25 (32.1)
Female	25 (19.7)	102 (80.3)	59 (46.5)	68 (53.5)	55 (43.3)	72 (56.7)	61 (48.0)	66 (52)	69 (54.3)	58 (45.7)	67 (52.8)	60 (47.2)

ALAD: Latin American Consensus of the Latin American Diabetes Association; IDF: International Diabetes Federation; NCEP-ATP III: National Cholesterol Education Program-Adult Treatment Panel III; AHA/NHLB: American Heart Association/National Heart, Lung, and Blood Institute Scientific Statement; AACE: American Association of Clinical Endocrinologists.

## Data Availability

Upon reasonable request to the corresponding author.
